# The Interaction of *Helicobacter pylori* with the Adherent Mucus Gel Layer Secreted by Polarized HT29-MTX-E12 Cells

**DOI:** 10.1371/journal.pone.0047300

**Published:** 2012-10-08

**Authors:** Brendan Dolan, Julie Naughton, Nicole Tegtmeyer, Felicity E. B. May, Marguerite Clyne

**Affiliations:** 1 University College Dublin, School of Medicine and Medical Science, Dublin, Ireland; 2 School of Biomolecular and Biomedical Science, Dublin, Ireland; 3 Conway Institute of Biomolecular and Biomedical Science, Belfield, Dublin, Ireland; 4 The National Childrens Research Centre, Dublin, Ireland; 5 Faculty of Medical Sciences, University of Newcastle upon Tyne, Newcastle upon Tyne, United Kingdom; University of Hyderabad, India

## Abstract

*Helicobacter pylori* colonises the gastric mucosa of humans. The majority of organisms live in mucus. These organisms are an important reservoir for infection of the underlying epithelium. Cell culture models for *H. pylori* infection do not normally possess a mucus layer. The interaction of *H. pylori* with TFF1, a member of the trefoil factor family found in gastric mucin, is mediated by lipopolysaccharide. To test the hypothesis that the interaction of *H. pylori* with TFF1 promotes mucus colonization we characterised the interaction of *H. pylori* with a mucus secreting cell line, HT29-MTX-E12. An isogenic mutant of *H. pylori* with truncated core oligosaccharides was produced and binding to TFF1 and ability to colonise HT29-MTX-E12 cells determined. The adherent mucus layer of HT29-MTX-E12 cells contained the gastric mucin MUC5AC and trefoil factors, TFF1 and TFF3. *H. pylori* was found within the mucus layer in discrete clusters and in close association with TFF1. It also interacted with the membrane bound mucin MUC1 and replicated when co-cultured with the cells. An isogenic mutant of *H. pylori* with a truncated LPS core did not interact with TFF1, and colonization of HT29-MTX-E12 cells was reduced compared to the wild-type strain (p<0.05). Preincubation of cells with wild type LPS but not with truncated LPS resulted in reduced colonization by *H. pylori.* These results demonstrate that the interaction of TFF1 with *H. pylori* is important for colonization of gastric mucus and the core oligosaccharide of *H. pylori* LPS is critical for this interaction to occur. HT29-MTX-E12 cells are a useful system with which to study the interaction of bacteria with mucosal surfaces and the effect of such interactions on mediating colonization.

## Introduction

The majority of bacterial infections in humans and animals result from pathogens colonizing the body via mucosal surfaces such as the gastrointestinal, respiratory and urinary tracts. *Helicobacter pylori* colonizes the gastric mucosa of humans and primates. Infection occurs early in life [1] and usually lasts for several decades unless eradicated by antimicrobials. *H. pylori* has been described as a paradigm for chronic infection of mucosal surfaces [2]. The majority of infecting bacteria live in the mucus layer that overlies the gastric epithelial cells [3] and colonization of experimental animals suggests that the organisms live close to the epithelial surface [4]. Organisms living in mucus act as a reservoir of bacteria which can interact with the underlying epithelium and consequently cause disease. Elucidation of the mechanisms that *H. pylori* uses to colonise mucosal surfaces could give us valuable insight into how pathogens overcome the barriers to infection such as the presence of a mucus layer.

*H. pylori* exhibits a very distinct tropism for the gastric mucin MUC5AC [5]. TFF1, a member of the trefoil factor family of proteins is co-expressed with MUC5AC in the stomach [6] and also interacts with MUC5AC [7]. TFF1 has been identified as a molecule that interacts with *H. pylori* [8]. We have previously identified low molecular weight or rough form (RF) lipopolysacharide (LPS) of *H. pylori* as a bacterial factor that interacts with TFF1 [9]. RF LPS contains the core oligosaccharide region of LPS but lacks the O antigen side chain. The aim of the present study was to test the hypothesis that the interaction of *H. pylori* with TFF1 promotes colonization of gastric mucus and that the core oligosaccharide of *H. pylori* LPS is the critical bacterial factor that mediates the interaction between *H. pylori* and TFF1.

A reliable *in vitro* system is required to study the role of bacterial colonization of mucus in promoting disease at the molecular and cellular level. Despite the obvious importance of understanding bacterial colonization and infection mechanisms, there are few model systems that allow for comprehensive studies on the role of the adherent mucus layer that covers many mammalian epithelial surfaces. HT29-MTX-E12 cells, a subclone of HT29-MTX cells which have been selected on the basis of tight junction formation, produce a mature adherent mucus gel layer when grown on transwell filters [10].

We have characterized mucin and trefoil protein expression in HT29-MTX-E12 cells and their adherent mucus layer and infected the cells with *H. pylori*. We present results which suggest that the interaction of *H. pylori* with TFF1 in the adherent mucus layer of HT29-MTX-E12 cells is important for colonization. Our results indicate that this cell model system has potential for studying the interaction of bacteria with mucus and the effect of such interactions on mediating bacterial colonization.

## Materials and Methods

### Cell culture

The HT29-MTX-E12 cell line, a mucus secreting subclone of the human colorectal adenocarcinoma cell line, HT29-MTX, was a generous gift from Professor Per Artursson, Uppsala University, Sweden. This clone was selected on the basis of tight junction formation and development of a mature adherent mucus layer [10]. HT29-MTX-E12 cells were maintained in Dulbecco's Modified Eagle Medium (DMEM; Lonza) supplemented with 10% (vol/vol) FBS, 1% (vol/vol) non-essential amino acids (Sigma) and 2 mM L-glutamax (Invitrogen). For experiments cells were grown for up to 21 days on Transwell filters 12 mm in diameter, with a 0.4 µm pore size (Millipore). Filters were seeded at a density of 1×10^5^ cells/filter and grown in DMEM F12 which also contained 100 U/ml penicillin (Sigma), 100 µg/ml streptomycin (Sigma) and 125 µg/ml amphotericin B (Sigma). Media was replaced every second day.

### Measurement of trans epithelial electrical resistance

The integrity of polarised HT29-MTX-E12 monolayers was checked by measurement of Trans Epithelial Electrical Resistance (TEER) using an EVOMAX meter and STX-2 probe (World Precision Instruments). TEER was measured at different time points over a 21 day culture period and expressed as Ω/cm^2^.

### Processing of cells for microscopy

Transwell filters with either infected or uninfected HT29-MTX-E12 cells were gently washed with sterile PBS, removed from their plastic supports and sandwiched between two thin pieces of chicken liver prior to mounting in Optimal Cutting Temperature (OCT) medium (BDH) as described by Keely *et* *al,* [11]. Sections (16 µm thick) of the OCT mounted cultures were cut using a cryostat (Leica) and collected onto polysine coated microscope slides. Sections on slides were allowed to air dry for 10–15 min and were then used immediately or stored at −20°C until use. Some cells were fixed in Carnoys fixative [12] and paraffin embedded. Sections were cut using a microtome and mounted on polysine slides. PAS staining of frozen sections of HT29-MTX-E12 cells was performed using the Haematoxylin/Periodic Acid Schiff Staining System (Sigma). Staining protocol was based on the manufacturer's guidelines for paraffin embedded tissue sections. Sections were covered with periodic acid solution for 5 min. Slides were washed in several changes of distilled water. Sections were covered with Schiff's reagent for 15 min and then washed under running tap water for 5 min. Finally sections were counterstained with Gill's hematoxylin solution for 90 sec and washed under running tap water. Excess water was removed from the stained slides and coverslips were mounted in DPX (BDH).

### Immunofluorescent staining of HT29-MTX-E12 cells

Frozen sections of cells were fixed on slides with 2% formalin for 10 min and permeablised with 0.2% (wt/vol) saponin (Sigma) in PBS for 10 min. Sections were incubated for 1 h in 1% (wt/vol) Bovine Serum Albumin (BSA; Sigma) and 10% (vol/vol) serum in PBS. Sections were then incubated overnight with antibodies against TFF1 [13], TFF2 [14], TFF3 [15], MUC1 (Ma522, Novocastra), MUC2 (Ccp58, Novocastra) MUC5AC C-terminus (45M1, Sigma), MUC5AC tandem repeat (CLH2, Santa Cruz), or *H. pylori* (Dako) at 4°C in a humidified atmosphere, washed with PBS and subsequently incubated with an anti-mouse or anti goat antibody conjugated to Alexafluor 488 (Invitrogen), or an anti rabbit antibody conjugated to either Alexafluor 594 or texas red. The secondary antibody solution included DAPI (Invitrogen) to counterstain the nuclei. Coverslips were mounted on the slides using Fluorescent Mounting Medium (Dako) and the sections were examined with a fluorescent microscope. In order to stain cells for the tight junction protein, ZO-1, the mucus layer was removed from the cells by treatment with N-acetyl cysteine as described previously [16]. After removal of the mucus, cells were fixed and permeabilised in cold methanol (−20°C) for 30 min prior to staining with anti ZO-1 N-terminus (Santa Cruz). Some cells were double labelled with the *H. pylori* antibody and another antibody (MUC5AC or TFF1 antibody). These samples were permeabilised as described above and incubated first with the *H. pylori* polyclonal antibody and secondary anti rabbit antibody and subsequently with the desired second primary antibody and secondary anti mouse antibody. Images were acquired with a Zeiss 510 UVMETA Confocal Microscope. Paraffin-embedded samples were deparaffinated and stained with antibodies against the trefoil proteins as described above following heat induced antigen retrieval. Slides were held in a pressure cooker for 3 min in antigen retrieval buffer, 10 mM sodium citrate buffer, pH6.0, containing 0.05% (vol/vol) Tween 20.

### Semi quantitative reverse transcriptase PCR

Total RNA was extracted from infected and uninfected cells with RNA Trizol reagent (Invitrogen) and was treated with amplification grade DNaseI (Invitrogen). Isolated RNA was quantified using a NanoDrop spectrophotometer. cDNA was synthesised from an equal amount of RNA (2 ug per reaction) using the Superscript First-Strand Synthesis System for RT-PCR (Invitrogen). Synthesised cDNA from 7, 14 and 21 day old uninfected and infected HT29-MTX-E12 cells was probed with primers specific for TFF1, TFF2, TFF3, MUC2, MUC5AC, MUC6 and β actin (Table 1). Primers and conditions used for the detection of trefoil peptide cDNA's were as described previously [17]. Primers and conditions used for the detection of MUC5AC and MUC6 cDNA's were as described by Paulsen *et* *al* [18]. Primers for the detection of MUC2 were a kind gift from Dr. Colm Reid (UCD School of Veterinary Medicine). The reaction mixture for MUC2 PCR's consisted of 30.1 µl water, 5 µl 10x reaction buffer (Invitrogen), 1.5 µl 50 mM MgCl_2_ (final concentration 1.5 mM; Invitrogen), 2 µl 10 mM dNTP mix (final concentration 0.4 mM; Invitrogen), 10 µl forward primer (final concentration 10 pmol), 5 µl reverse primer (final concentration 10 pmol), 0.4 µl Platinum Taq polymerase (2 units; Invitrogen) and 1 µl cDNA. The PCR cycle consisted of an initial incubation at 94°C for 5 min followed by 35 cycles of 94°C for 1 min, 54°C for 1 min and 72°C for 90 sec. The cycle concluded with a final incubation of 72°C for 10 min. The detection of cDNA corresponding to the β actin mRNA was used as a positive control for each PCR. Each reaction contained 30.1 µl autoclaved deionised water, 5 µl 10x PCR buffer (Invitrogen), 1.5 µl 50 mM MgCl_2_ (final concentration 1.5 mM; Invitrogen), 2 µl 10 mM dNTP mix (final concentration 0.4 mM; Invitrogen), 5 µl forward primer (10 pmol; MWG-Biotech), 5 µl reverse primer (10 pmol; MWG-Biotech), 0.4 µl Platinum Taq polymerase (2 units; Invitrogen) and 1 µl cDNA. The PCR cycle consisted of 94°C for 7 min followed by 35 cycles of 94°C for 30 sec, 55°C for 30 sec and 72°C for 1 min. The cycle concluded with a final extension time of 7 min. All PCR reactions were carried out in 200 µl nuclease free reaction tubes (Sarstedt) in a MWG-Biotech Primus 96 thermocycler. After amplification PCR products were examined by agarose gel electrophoresis. Quantification of RT PCR products was assessed by densitometry using the GelDoc-IT Imaging System and Labworks Analysis software (UVP, Cambridge, UK). Results are an average of three densitometry readings relative to expression of the house keeping gene, β actin.

**Table 1:** Oligonucleotide primers used for reverse transcriptase PCR.

	Forward Primer	Reverse Primer	Reference
**TFF1**	TTTGGAGCAGAGAGGAGG	TTGAGTAGTCAAAGTCAGAGCAG	[17]
**TFF2**	AGTGAGAAACCCTCCCCC	AACACCCGGTGAGCCAG	[17]
**TFF3**	GTGCCAGCCAAGGACAG	CGTTAAGACATCAGGCTCCAG	[17]
**MUC2**	CACCCCTTCCTCAACTACCA	CGGAGTTGGGTTCTCTGTG	This study
**MUC5AC**	CCCAAGGAGAACCTCCCATAT	CCASGCGTCATTCCTGAG	[18]
**MUC6**	CAGCAGGAGGAGATCACGTTCAAG	GTGGGTGTTTTCCTGTCTGTCATC	[18]
**β Actin**	GCTATCCCTGTACGCCTCTG	CTCCTTCTGCATCCTGTCGG	[32]

### Bacterial strains and culture conditions

*H. pylori* strains PU4, P12 and PA4 were used in this study. Frozen *H. pylori* stocks were recovered onto Columbia agar (Oxoid) containing 7% (vol/vol) defibrinated horse blood. *H. pylori* isogenic mutant strains were recovered onto media supplemented with kanamycin sulphate (Fluka) to a final concentration of 10 µg/ml. *H. pylori* strains were cultured at 37°C in gas jars (Oxoid; BBL) under microaerophillic conditions generated by CampyGen gas packs (Oxoid).

### Cloning and mutagenesis of *H. pylori*

Isogenic mutants PA4ΔHP1191 and P12ΔHP1191 were constructed by insertion of a kanamycin resistance gene cassette (AphA3, 1.4 kb SmaI fragment) in the cloned HP1191 gene according to a standard protocol [19]. Briefly, the gene was amplified by polymerase chain reaction (PCR) using primers 577F 5′ATGAGCGTAAATGCACCCAAACGC and 577R 5′CTCTTCTAAAAGAGTGTGAGCGGC (1,047 bp). The PCR product was cloned in the pGEM-T vector (Promega). After mutagenesis by insertion of the AphA-3 cassette in the genes, 5 µg of supercoiled plasmid DNA was added to 1×10^8^ bacteria per ml in brain heart infusion (BHI) medium. After incubation for 6 h, bacteria were grown on agar plates containing 8 µg per ml kanamycin to select for kanamycin-resistant transformants obtained after 4–5 days of incubation. Correct integration of the AphA-3 cassette into the *H. pylori* chromosome by double cross-over recombination was confirmed by PCR.

### Infection assays

Cells were grown on transwell filters as described above and 24 h before infection antibiotic containing media was removed from the cells, monolayers were gently rinsed with antibiotic free media and cells were incubated in antibiotic free media overnight. Bacteria were grown as described above and used to infect HT29-MTX-E12 cells. *H. pylori* were harvested from Columbia blood agar into BHI broth at pH 5.0. Media in the lower chamber of the Transwell was replaced with 1.5 ml of sterile antibiotic free DMEM supplemented with 10% (vol/vol) FBS. Since *H. pylori* cannot survive in DMEM media in the upper chamber of the Transwell was replaced with 100 µl of sterile BHI broth, pH 5.0. These conditions have been shown previously to be able to support *H. pylori* growth and adherence to polarized epithelial cells without any adverse affects on the epithelial cells [20]. 50 µl of *H. pylori* suspension, OD_600_ = 0.4 was then added to the upper chamber of the Transwell. Infected cell cultures were incubated at 37°C in gas jars under microaerophillic conditions generated by CampyGen gas packs for up to 24 h. Bacterial colonization of the cells with *H. pylori* was assessed by microscopy as described above or by determination of the number of CFU associated with the cells. Infected cultures were gently washed with sterile PBS on both sides of the Transwell. Cells were harvested using trypsin EDTA as described previously [20]. Serial dilutions of the trypsinised cells were plated out in triplicate onto Columbia blood agar plates and incubated at 37°C in gas jars under microaerophillic conditions. Colonies were enumerated after 4–5 days incubation. For blocking experiments with LPS, purified LPS at a concentration of 100 µg/ml from either *H. pylori* strain PA4 or strain PA4Δ1191 was added to the apical surface of cells growing on Transwell filters for 4 h. Cells with and without LPS were infected with strain PA4 as described above and the number of CFU associated with the cells after a 2 hour incubation period was determined.

### Isolation of LPS from *H. pylori*

LPS was extracted from *H. pylori* using a mini phenol-water extraction procedure as described by Prendergast *et* *al* [21].

### Preparation of bacterial lysates

Bacteria were harvested from blood agar plates and resuspended in PBS. The bacterial suspension was then heated to 100°C for 10 min in NuPAGE LDS sample buffer (4X) (Invitrogen).

### Preparation of HT29-MTX-E12 cell lysates

Lysates of HT29-MTX-E12 cells grown for 21 days on Transwell filters were prepared using a commercially available mammalian cell lysis extraction reagent (CelLytic™ M, Sigma) according to the manufacturers instructions.

### Electrophoresis and overlay blotting

Samples of bacterial whole cell lysates, and purified LPS were electrophoresed on NuPAGE® Novex Bis-Tris gels containing 12% acrylamide (Invitrogen). Proteins were transferred to PVDF membrane (Millipore) overnight at 75 mA, 4°C in a wet blotter (BioRad). PVDF membranes were blocked with Marvel (3% wt/vol) in PBS Tween (0.05% vol/vol) for 1 h and then biotinylated TFF1 dimer (5 µg/ml) was added in blocking buffer overnight at 4°C. Blots were washed 3×10 min in PBS Tween (0.05%vvol/vol). Streptavidin-peroxidase was used to detect reactive bands by enhanced chemiluminescence (GE). For detection of the interaction of TFF1 expressed in HT29-MTX-E12 cells with *H. pylori* LPS blots were overlaid with a whole cell lysate of HT29-MTX-E12 cells for 2 h at 37°C. After washing the filter was incubated with a monoclonal TFF1 antibody followed by horseradish peroxidase-conjugated rabbit anti-mouse antibody. Reactive bands were detected by enhanced chemiluminescence.

### Statistical analysis

Experiments were conducted on at least three separate occasions in triplicates. Results are presented as the means ± standard deviations (error bars) of replicate experiments. Graphs were drawn using either Microsoft Excel or Prism Graph Pad. The Student *t* test was used to estimate statistical significance. A *P* value of <0.05 was considered significant.

## Results

In order to assess the suitability of HT29-MTX-E12 cells for infection studies with *H. pylori* and specifically to study the interaction of the organism with trefoil peptides in mucus we characterized tight junction formation and mucin and trefoil peptide expression in the cells.

### Tight junction formation

**Figure 1:**
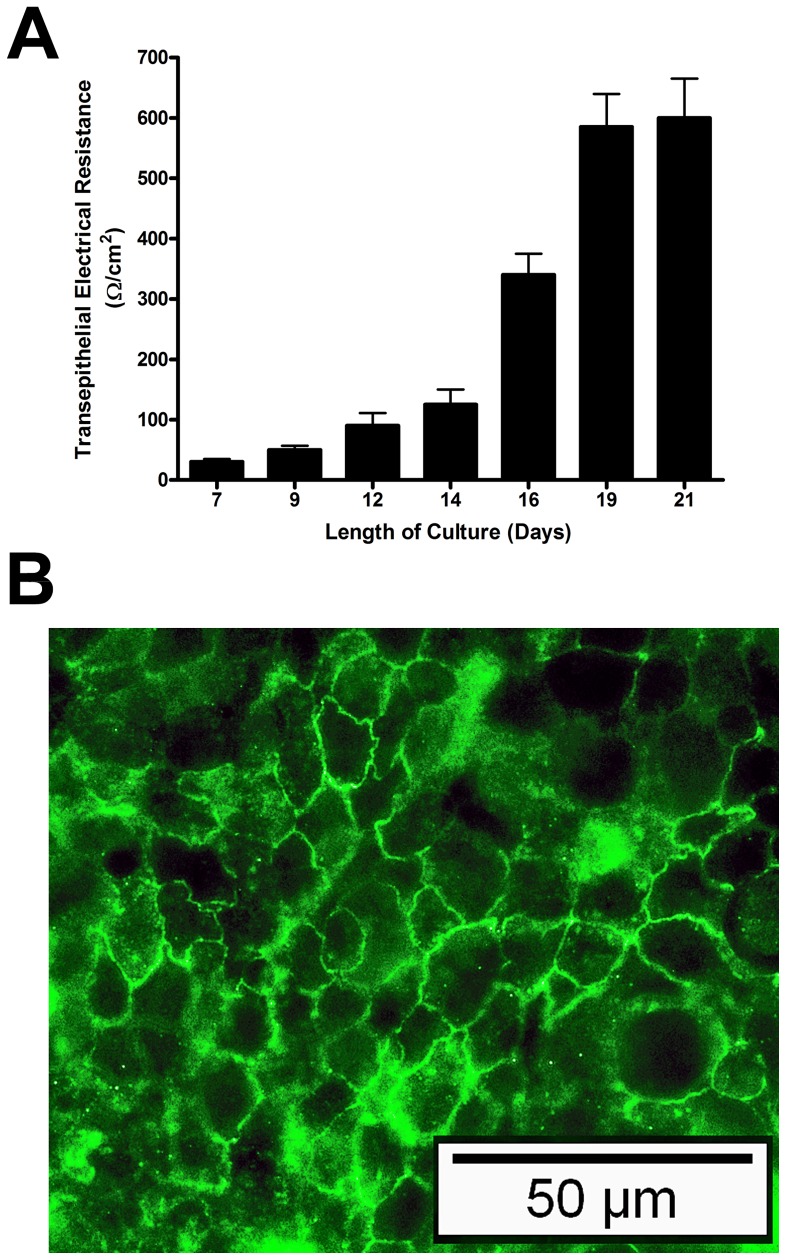
Tight junction formation in HT29-MTX-E12 cells over a 21 day period of differentiation. (A) Transepithelial electrical resistance values (Ω/cm^2^) were measured at different timepoints throughout a 21 day period of differentiation. (B) ZO-1 staining of HT29-MTX-E12 cells at day 21. Error bars indicate mean ± Standard Deviation of three separate experiments.

HT29-MTX-E12 cells were grown for 21 days on Transwell filters. The formation of tight junctions at different time points was measured by monitoring the transepithelial electrical resistance (TEER). As reported previously for HT29-MTX-E12 cells [10] the TEER increased steadily over time and reached a stable value of approximately 600 Ω/cm^2^ by days 19–21 (Figure 1A). The location of the tight junction associated protein ZO-1 around the cell membranes confirmed the presence of tight junctions between neighbouring cells (Figure 1B).

### Expression and cellular localization of mucins and trefoil peptides

**Figure 2:**
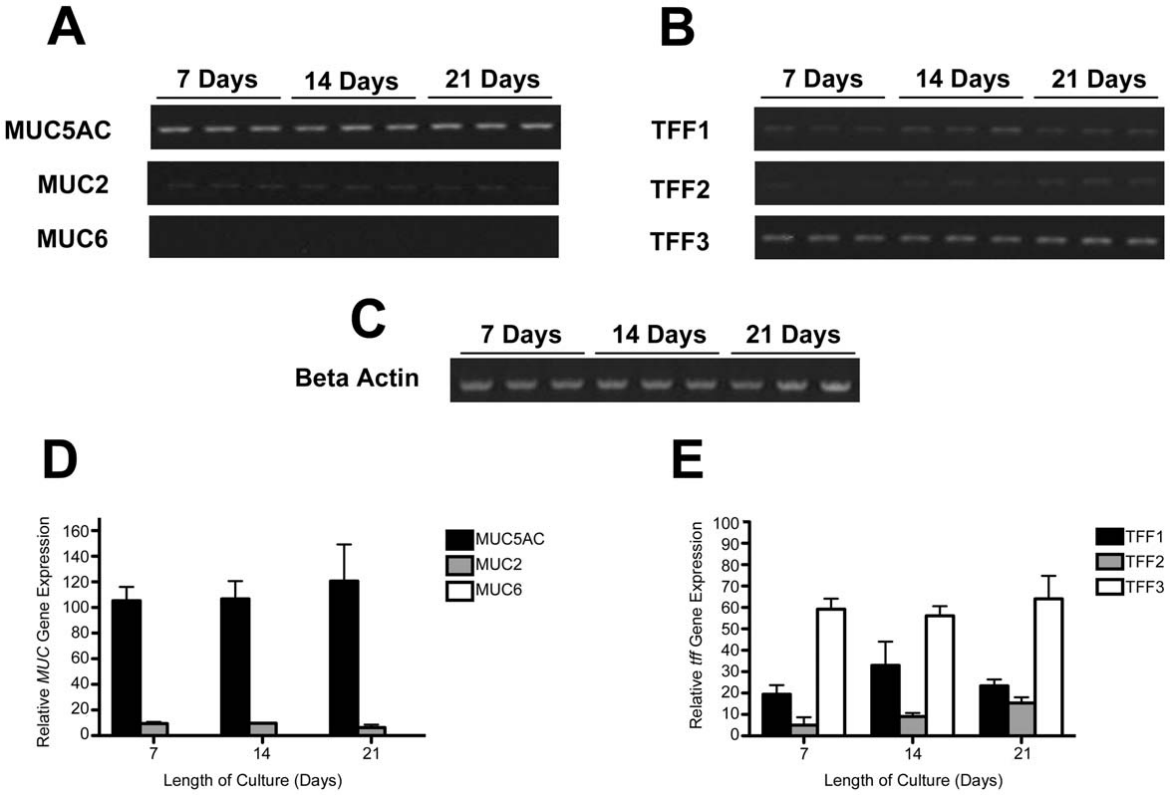
Mucin and trefoil peptide gene expression in HT29-MTX-E12 cells. HT29-MTX-E12 cells were grown on transwell filters for up to 21 days. Reverse transcription PCR was used to assess mucin (A and D) and trefoil peptide (B and E) mRNA expression at 7, 14 and 21 day timepoints. Expression is relative to expression of the house keeping gene, β actin (C). Error bars indicate mean ± Standard Deviation of three separate experiments.

Expression of the gastric mucins MUC5AC and MUC6, the intestinal mucin MUC2 and the trefoil proteins TFF1, TFF2 and TFF3 in HT29-MTX-E12 cells was assessed by reverse transcription PCR. MUC5AC mRNA expression was detected readily in HT29-MTX-E12 cells at days 7, 14 and 21. MUC2 mRNA was also present. No expression of MUC6 mRNA was detected at any time during the 21 day culture period (Figure 2A and 2D). TFF1, TFF2 and TFF3 mRNA expression was detected at all times during culture (Figure 2B and 2E). There were no obvious differences in expression of the mRNAs during differentiation of the HT29-MTX-E12 cell monolayer.

**Figure 3:**
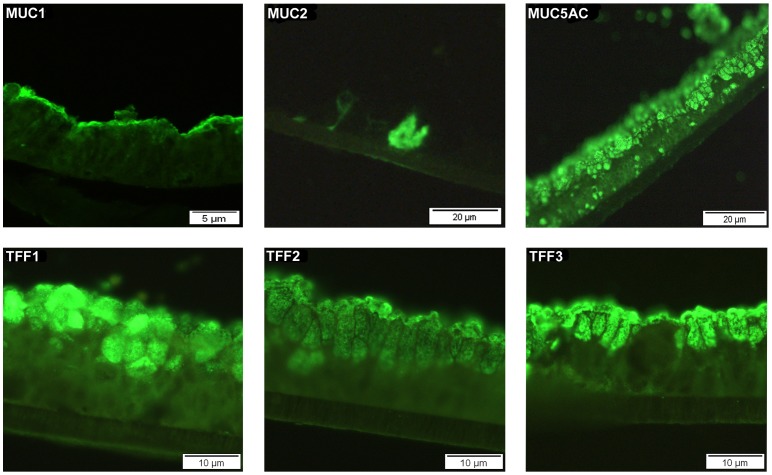
Cellular localization of MUC1, MUC2, MUC5AC, TFF1, TFF2 and TFF3 in HT29-MTX-E12 cells. Paraffin embedded HT29-MTX-E12 cells grown on transwell filters for 21 days were stained with specific antibodies for MUC1, MUC2, MUC5AC, TFF1, TFF2 and TFF3.

The cellular location of the expressed mucin and trefoil proteins was investigated in HT29-MTX-E12 cells grown for 21 days by immunofluoresence with specific antibodies against MUC1, MUC2, MUC5AC, TFF1, TFF2 and TFF3. The membrane bound mucin MUC1 was localised on the apical surface of the cells. MUC2 was detected intracellularly in occasional cells. There was clear apical, intracellular expression of MUC5AC, TFF1, TFF2 and TFF3 (Figure 3). Particularly strong expression of TFF1 was detected in the mucus vesicles on the surface of the HT29-MTX-E12 cells.

### Composition of the adherent mucus layer

**Figure 4:**
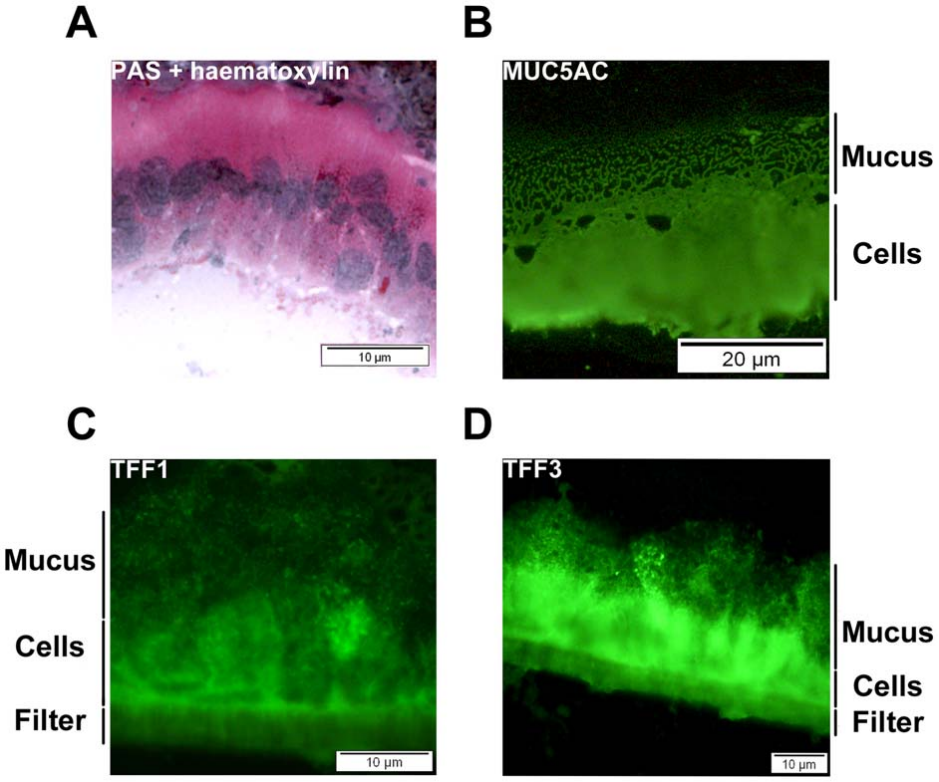
Characterization of the extracellular mucus layer of HT29-MTX-E12 cells . Frozen sections of 21 day old HT29-MTX-E12 cells were stained with (A) PAS and haematoxylin or with specific antibodies against (B) MUC5AC, and trefoil peptides, (C) TFF1, and (D) TFF3. An adherent extracellular mucus layer could be visualised using PAS and haematoxylin staining. MU5AC, TFF1 and TFF3 were detected in this extracellular mucus.

The adherent mucus layer of HT29-MTX-E12 cells is largely destroyed during formalin fixation and paraffin embedding. To preserve the mucus layer, polarised HT29-MTX-E12 cells grown on transwell filters for 21 days were wrapped in liver, embedded in OCT and frozen in liquid nitrogen. Frozen cells were sectioned using a cryostat and stained with PAS and haematoxylin or with specific antibodies. Incubation of frozen sections with PAS and haematoxylin demonstrated that the cells had formed a thick adherent mucus gel layer. The presence of the mucin MUC5AC and the trefoil proteins TFF1 and TFF3 in the adherent mucus layer were confirmed by immunofluoresence with specific antibodies (Figure 4). MUC2 and TFF2 were not detected in the adherent mucus layer by immunofluorescent microscopy. This result was not surprising for MUC2 as we only detected occasional cells expressing this mucin intracellularly. There was however good intracellular expression of TFF2. The reason why we did not detect extracellular TFF2 however may be due either to lack of secretion of this protein from the cells or possibly the antibody may require heat induced antigen retrieval of the cells to work.

### Infection of HT29-MTX-E12 cells by *H. pylori*

**Figure 5:**
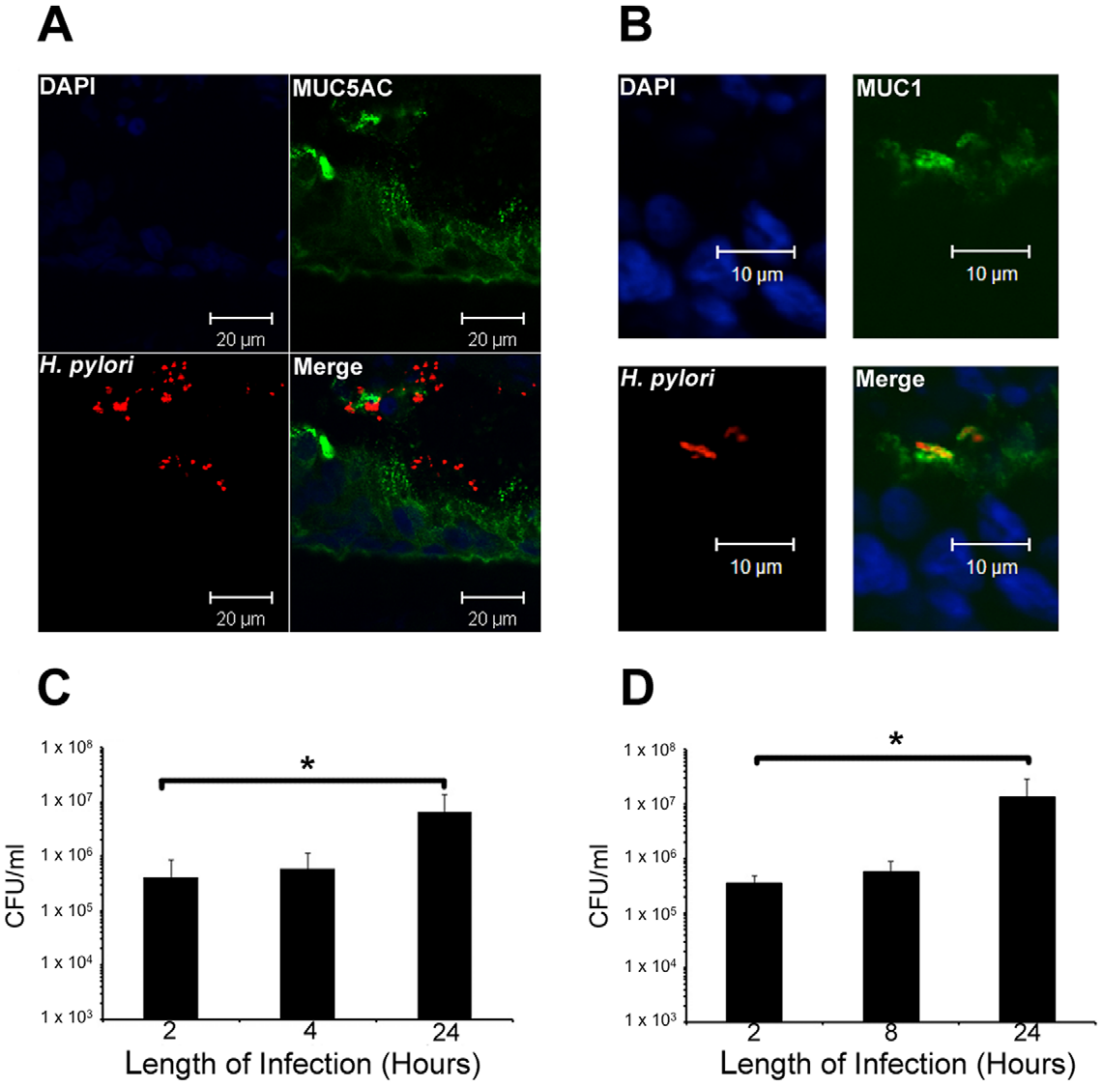
Colonization of HT29-MTX-E12 cells by *H. pylori* . (A and B) Immunofluorescent microscopy was used to visualise *H. pylori* colonizing HT29-MTX-E12 cells. *H. pylori* strain PU4 was used to infect HT29-MTX-E12 cells for 24 h after which time frozen sections were stained with specific antibodies against *H. pylori* (red) and MUC5AC or MUC1 (green). DAPI (blue) staining was used to visualise cell nuclei. Organisms were found in discrete foci or clusters throughout the mucus layer and close to the epithelial cells. (C) Colonization of 21 day old HT29-MTX-E12cells with *H. pylori* strain PU4 over a 24 h period of infection. There were significantly more organisms associated with the cells after 24 h of infection compared with 2 h or 4 h of infection, (* p≤0.05). (D) Replication of *H. pylori* strain PU4 co-cultured with 21 day old HT29-MTX-E12 cells over a 24 h period. Cells were infected with *H. pylori* for 2 h after which time the cells were washed thoroughly to remove any non adherent bacteria. Cells were incubated for a further 22 h and the total number of bacteria associated with the cells was determined. The number of bacteria associated with the cells was significantly increased at the 24 h time point compared to the 2 h time point, indicating that the organism reproduced while associated with the cells (*p≤0.05). Error bars indicate mean ± Standard Deviation of three separate experiments.

All our infection studies were with HT29-MTX-E12 cells after 21 days of culture because at this time they had formed tight junctions and secreted a mature adherent mucus layer which contained the mucin MUC5AC and trefoil protein TFF1. Cells were infected with *H. pylori* for up to 24 h. Single *H. pylori* organisms could be visualised clearly by immunofluorescent confocal microscopy in frozen sections of HT29-MTX-E12 cells. The bacteria were found in discrete foci or clusters within the MUC5AC mucus layer (Figure 5a). Bacteria were also found at the apical surface of the underlying epithelial cells interacting with the membrane bound mucin MUC1 (Figure 5B). The infected cells were lysed and the number of CFU of bacteria recovered after different times of infection was measured. This quantifies the total number of bacteria in the adherent mucus layer and associated directly with the cells. Two hours post infection, 4.12×10^5^ CFU/ml were recovered and by 24 h this had increased 16-fold to 6.5x10^6^ CFU/ml, (p≤0.05), (Figure 5C). The ability of *H. pylori* to replicate when co-cultured with HT29-MTX-E12 cells was assessed by washing cells to remove any non colonizing bacteria 2 h after infection, replacing the medium and then measuring the number of CFU/ml present in cell lysates at different time points. Two hours after infection, 3.53×10^5^ CFU/ml were associated with HT29-MTX-E12 cells. By 22 h after removal of the bacteria that had not colonised the HT29-MTX-E12 cells, the number of bacteria had increased nearly 25-fold to 8.17×10^6^ CFU/ml (p≤0.05) (Figure 5D). This increase in bacterial load in the adherent mucus and associated with the cells must be due to bacterial replication as there were no bacteria in the medium available for further colonization after the 2 h timepoint. These results demonstrate that *H. pylori* colonizes HT29-MTX-E12 cells and that the bacteria undergo cell division with a cell doubling time of approximately 4–5 h.

### Interaction of *H. pylori* with TFF1 in HT29-MTX-E12 cells

**Figure 6:**
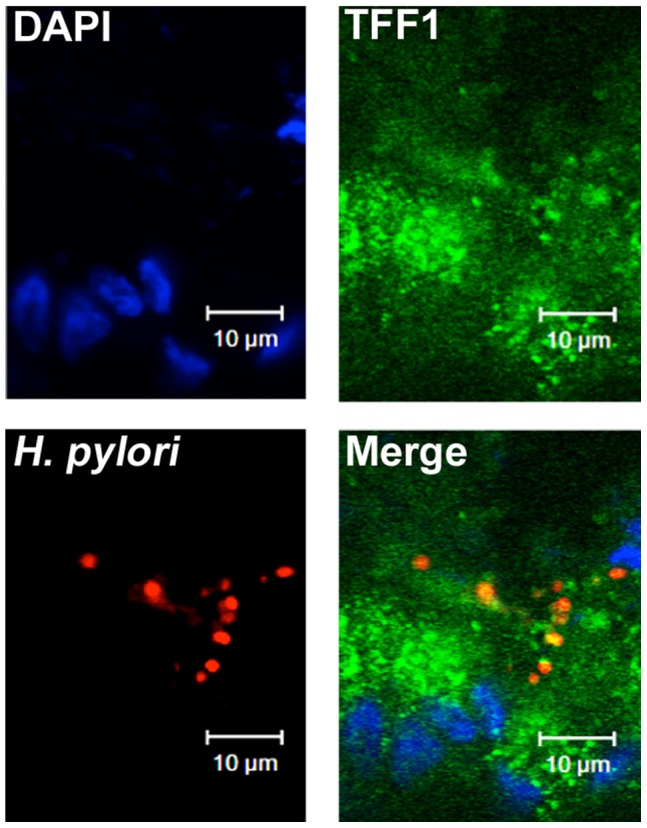
The interaction of *H. pylori* with TFF1 in HT29-MTX-E12 cells. HT29-MTX-E12 cells grown for 21 days on transwell filters were infected with *H. pylori* for 24 h. Infected cells were stained with antibodies against *H. pylori* (red) and against TFF1 (green). DAPI staining (blue) was used to visualise cell nuclei.

As described above, we showed that the extracellular mucus layer produced by HT29-MTX-E12 cells contains TFF1 and that after infection *H. pylori* organisms can be visualised within the mucus layer. We investigated if the bacteria might interact with TFF1 in this HT29-MTX-E12 cell model (Figure 6). TFF1 immunofluorescence was discrete and the *H. pylori* foci co-localised with areas of TFF1 expression in the HT29-MTX-E12 mucus layer which is consistent with there being an interaction between TFF1 and *H. pylori* in adherent mucus.

### Interaction of TFF1 from HT29-MTX-E12 cells with *H. pylori* LPS

**Figure 7:**
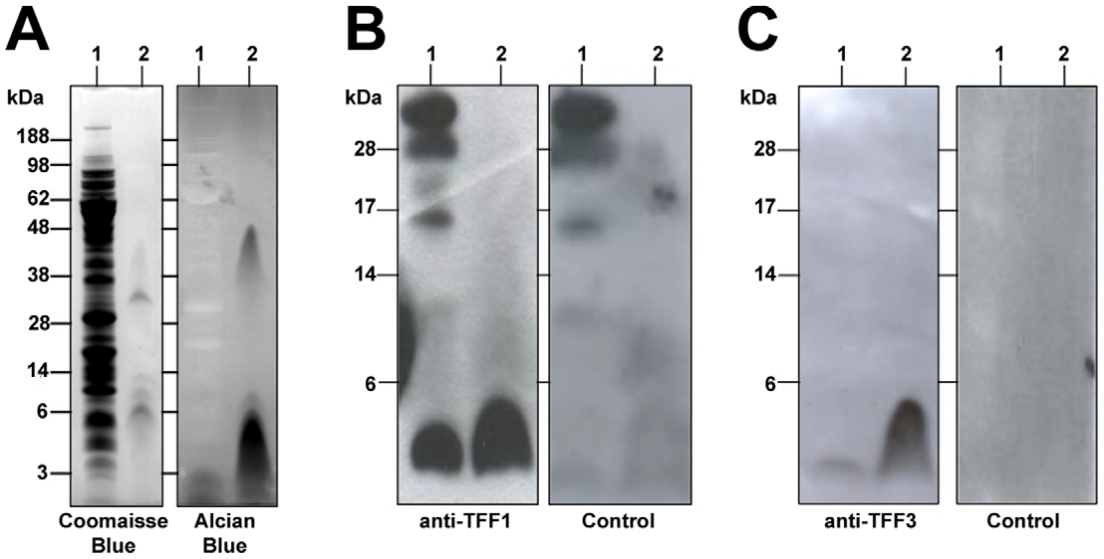
TFF1 in HT29-MTX-E12 cells interacts with *H. pylori* LPS. A *H. pylori* total cell lysate (Lane 1)and purified LPS (Lane 2) were electrophoresed on SDS PAGE and (A) stained with Coomassie blue which stains proteins or alcian blue which stains carbohydrate moieties or (B and C) transferred onto PVDF membrane and probed with a total cell lysate made from uninfected HT29-MTX-E12 cells. The filter was then probed with either an antibody to TFF1 (B) or an antibody to TFF3 (C) and a horseradish peroxidase conjugated secondary antibody. Strong TFF1 binding and weak TFF3 binding to low molecular weight RF LPS of *H. pylori* was detected using the ECL system from Amersham. Identical overlay blots (controls) were probed with just the secondary antibody. This confirmed that only the low molecular weight (6 kDa) band was specific for TFF1 and TFF3 binding.

We tested if TFF1 or TFF3 produced by HT29-MTX-E12 cells interacts with *H. pylori* LPS as has been shown previously with recombinant trefoil peptides [9]. Total *H. pylori* cell lysate or purified LPS were transferred onto PVDF membranes and incubated with HT29-MTX-E12 cell lysate. The membranes were then incubated with specific antibodies against either TFF1 or TFF3 to detect protein that had bound to constituents of *H. pylori* immobilised on the membranes. Strong binding of TFF1 to the low molecular weight LPS present in whole *H. pylori* lysate and to purified *H. pylori* LPS was detected indicating that TFF1 produced by HT29-MTX-E12 cells interacts with *H. pylori* rough form LPS (Figure 7A and 7B). There was some interaction of TFF3 with purified LPS of *H. pylori* but the interaction with the cell lysate was barely detectable (Figure 7C). These results suggest that *H. pylori* can interact with TFF1 in HT29-MTX-E12 cells and that there is also some interaction, albeit not as strong as the interaction with TFF1, with TFF3 and that the interaction of *H. pylori* with TFF1 and TFF3 in HT29-MTX-E12 cells is mediated probably by *H. pylori* LPS.

*H. pylori* isogenic mutants with truncated LPS are unable to interact with TFF1 and exhibit reduced colonization of HT29-MTX-E12 cells.

**Figure 8:**
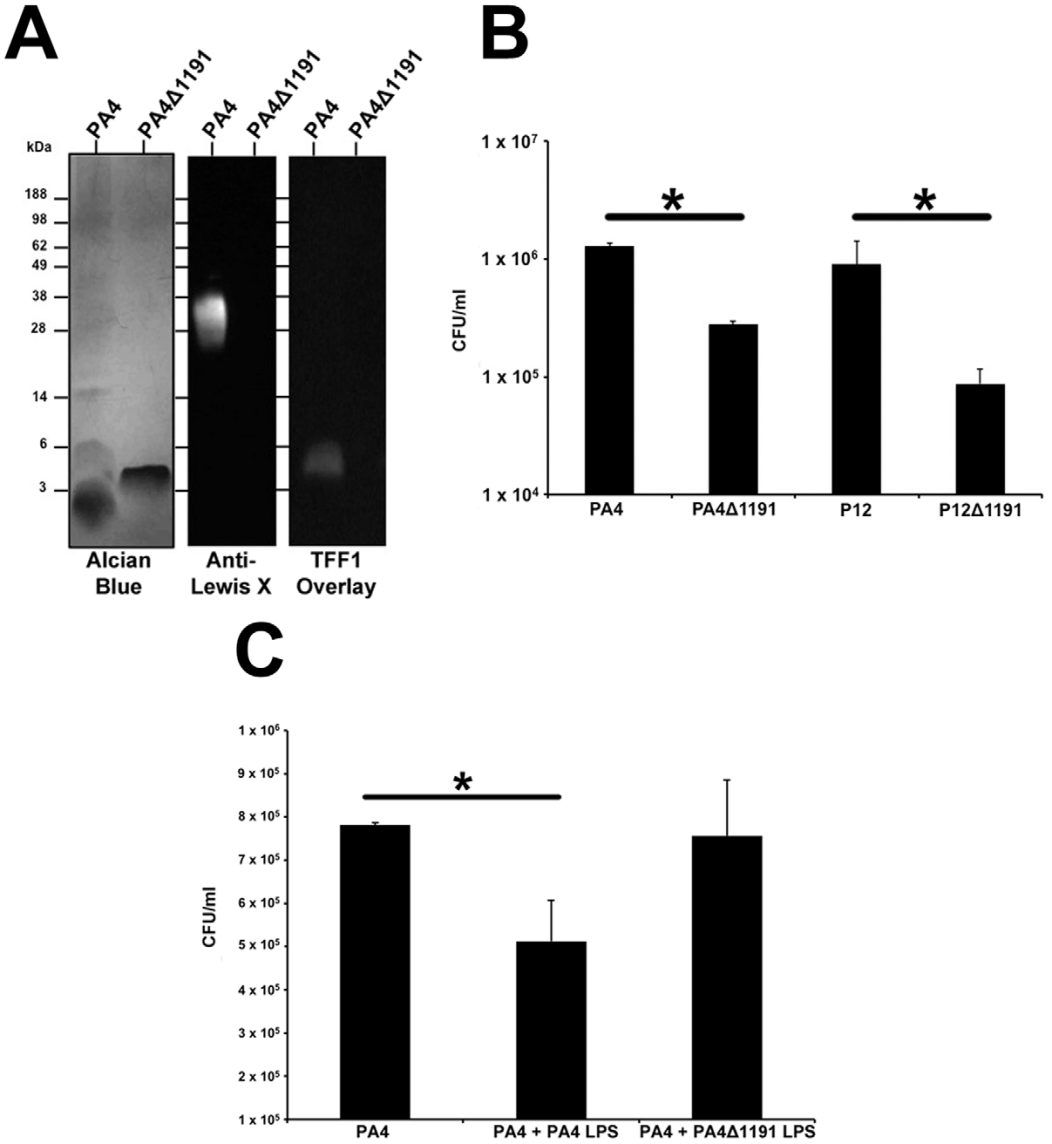
*H. pylori* mutants producing truncated LPS do not interact with TFF1 and exhibit reduced colonization of HT29-MTX-E12 cells. (A) LPS from *H. pylori* strains PA4 and PA4ΔHP1191 was run on SDS PAGE and stained with alcian blue or blotted onto PVDF membrane and probed with either anti Lewis x antibody or with dimeric TFF1. (B) *H. pylori* strains PA4ΔHP1191 and P12ΔHP1191 exhibited significantly reduced colonization of HT29-MTX-E12 cells compared with WT parental strains (*p<0.05). (C) Incubation of cells with purified LPS from wild type strain PA4 but not with LPS from strain PA4ΔHP1191 reduced subsequent colonization of the cells with strain PA4 (*p<0.05).

In order to further test the importance of *H. pylori* LPS interaction with TFF1 in mediating *H. pylori* colonization of adherent mucus, we constructed isogenic mutants of *H. pylori* that produce truncated LPS core oligosaccharides. HP1191, encodes an LD-heptosyltransferase which has been shown to be involved in the biosynthesis of the inner core region of the oligosaccharide portion of LPS of *H. pylori* strain 26695 and insertional inactivation of HP1191 resulted in the production of LPS with a severely truncated core oligosaccharide [22]. We constructed *H. pylori* strain PA4ΔHP1191 and tested the ability of TFF1 to bind to the LPS of PA4 wild-type and PA4ΔHP1191. Alcian blue staining of LPS on SDS PAGE gels and western blotting with antibodies against Lewis x demonstrated that compared to LPS from the wild type parental strain PA4, the LPS of PA4ΔHP1191 was truncated and no longer expressed O antigen side chain as evidenced by lack of reactivity with antibodies against Lewis x (Figure 8A). Dimeric TFF1 bound to LPS from PA4 but not to LPS from PA4ΔHP1191 (Figure 8A) which indicates that it is the core oligosaccharide portion of *H. pylori* LPS that interacts with TFF1. We then assessed colonization of HT29-MTX-E12 cells by wild type strains PA4 and P12 and mutants PA4ΔHP1191 and P12ΔHP1191. The level of colonization by both mutants was significantly reduced compared to colonization by the wild-type parental strains (p<0.05) (Figure 8B). Incubation of cells with LPS from wild type strain PA4 but not with LPS from strain PA4ΔHP1191 and subsequent colonization of cells with strain PA4 resulted in reduced bacterial colonization compared to cells infected in the absence of LPS (p<0.05) (Figure 8C).

## Discussion

To date the *in vitro* cell culture models used to study the interaction of bacteria with epithelial surfaces have been confined largely to epithelial cell lines which do not produce an adherent mucus gel layer. This has limited our understanding of the interactions between bacteria and mucus which are an important first step in colonisation. In order to assess the role of TFF1 in mediating *H. pylori* colonization of adherent mucus we had to identify a suitable *in vitro* model system. HT29-MTX-E12 cells grew in a similar way to that described previously [10]. During the 21 days in culture, the TEER increased which is indicative of tight junction formation and cell polarization. It has been shown that this cell line differentiates into mature mucus secretory cells [10]. PAS-Schiff staining confirmed that after 21 days of culture, HT29-MTX-E12 cells had formed an adherent mucus layer.

*H. pylori* was able to colonize HT29-MTX-E12 cells and bacteria could be visualized in the mucus layers of infected cells and close to the epithelial surface of the cells. *H. pylori* could be visualised within the mucus layer usually occurring in discrete clusters throughout the mucus and the organism was able to reproduce when co-cultured with the cells. *H. pylori* has also been shown to reproduce and form microcolonies over the intercellular junctions at the apical surface of polarized MDCK cells [23]. In addition a recent study has reported that the interaction of *H. pylori* with oligosaccharides present on mucins can promote bacterial proliferation [24]. The closely related organism *Campylobacter jejuni* can also colonize and reproduce when co-cultured with HT29-MTX-E12 cells. *C. jejuni* was scattered throughout the mucus layer, inside the cells, between the cells and at the basolateral surface [16]. This was in stark contrast to *H. pylori*, where the organisms were found in discrete foci within the mucus layer. This suggests that HT29-MTX-E12 cells may be a useful model to discriminate between different patterns of infection that occur with various pathogens.

The predominant secreted mucin detected in the mucus layer was MUC5AC with only very small amounts of MUC2 produced at any time point. MUC5AC, which is produced *in vivo* in the stomach, was localized to both the mucus layer and mucin granules whereas the intestinal mucin, MUC2 was only detected intracellularly in a small number of cells. There was prominent immuno-reaction with the membrane bound mucin MUC1 which is exposed on the apical surface of cells embedded in the plasma membrane and does not form a mucus gel. *H. pylori* binds directly to purified MUC5AC and MUC1 mucins [25], [26] and MUC1 has been shown to be important in limiting infection by *Helicobacter* and *Campylobacter* in a mouse model of infection [27], [28]. In this study *H. pylori* interacted directly with MUC1 on the apical surface of the cells. Interestingly recent work has shown that over expression of MUC1 counter regulated *H. pylori* induced gastric inflammation [29]. A follow on study has demonstrated that the interaction of *H. pylori* with MUC1 blocks *H. pylori* stimulated β-catenin nuclear translocation and attenuates IL-8 production and neutrophil gastric inflammation [30]. The speculation by the authors that augmented expression of MUC1 could be used for treatment of *H. pylori* gastritis highlights the need for model systems that express adherent mucus layers and appropriate mucins in order to test such hypotheses. The expression of MUC5AC and MUC1 by HT29-MTX-E12 cells suggest that these cells may be a useful system to further elucidate how *H. pylori* interacts with these mucins and the importance of the interactions for the development of disease and/or host defence.

Trefoil proteins are an integral structural component of mucus gels and TFF1 has been shown to be concentrated in the normal human gastric adherent mucus gel layer [31]. RT-PCR confirmed mRNA expression of all three trefoil factor family members in HT29-MTX-E12 cells. TFF1, TFF2 and TFF3 proteins were detected intracellularly concentrated in the mucus granules of the differentiated HT29-MTX-E12 cells and staining of TFF1 and TFF3 was evident in the adherent mucus gel layer. We could not detect TFF2 expression in the adherent mucus gel layer by immunostaining but as previously mentioned we think this may be due to the fact that our antibody may not work with frozen sections. Although HT29-MTX-E12 cells are a colonic cell line they do have a gastric phenotype. The predominant mucin expressed and secreted is MUC5AC and they also express the gastric trefoil proteins TFF1 and TFF2. These findings suggested to us that these cells were likely to be suitable to assess the importance of the interaction of *H. pylori* with trefoil peptides in mediating colonization of mucus.

*H. pylori* was found in close association with TFF1 in HT29-MTX-E12 cells. In addition overlay blotting studies showed that TFF1 produced by E12 cells could interact with *H. pylori* LPS. Previous work with gastric tissue sections has indicated that the interaction of TFF1 with LPS plays an important role in mediating *H. pylori* colonization of the gastric mucosa [9]. To elucidate further the biological relevance of the *H. pylori* LPS interaction with TFF1, we constructed isogenic mutants of *H. pylori* that produce truncated forms of the core oligosaccharide of *H. pylori* LPS. Low molecular weight LPS of *H. pylori* has been shown to mediate binding of *H. pylori* to TFF1 [9]. *H. pylori* PA4ΔHP1191 was unable to bind to TFF1 and exhibited reduced colonization of HT29-MTX-E12 cells compared to the wild type strain. Reduced colonization was also demonstrated with P12ΔHP1191. In a study from another laboratory a Hp1191 mutant created in *H. pylori* strain 26695 was able to invade AGS cells to a comparable level compared to the wild type strain [22]. This indicates that the decrease in colonization of HT29-MTX-E12 cells by PA4ΔHP1191 and P12ΔHP1191 is most likely due to lack of ability to interact with TFF1 in the mucus layer rather than an inability to interact with epithelial surfaces. The presence of LPS from wild type bacteria but not truncated LPS from PA4ΔHP1191 reduced colonization by wild type bacteria. Together these results demonstrate the likely importance of the interaction of TFF1 with *H. pylori* in the gastric mucosa and the role of the core oligosaccharide of *H. pylori* LPS in mediating that interaction.

In summary these results suggest that HT29-MTX-E12 cells are a useful model for the study of how mucosal pathogens interact with mucus gels. The finding that TFF1 may be important in mediating *H. pylori* colonization of the gastric mucosa suggests that we need to consider carefully the role of non mucin components of mucus in mediating infection of mucosal surfaces. TFF1 is present in the gastric mucosa bound covalently to a protein known as TFIZ1 or gastrokine 2 [13]. In addition, it has been shown that TFF1 interacts directly with MUC5AC *in vivo* [7]. We do not know if the interaction of *H. pylori* with TFF1 has an influence on TFF1 binding to other molecules and if it does what the biological consequences are. Studies now need to address these possibilities.
